# Benefits and Trade-Offs of Dairy System Changes Aimed at Reducing Nitrate Leaching

**DOI:** 10.3390/ani9121158

**Published:** 2019-12-17

**Authors:** Pierre Beukes, Alvaro Romera, Kathryn Hutchinson, Tony van der Weerden, Cecile de Klein, Dawn Dalley, David Chapman, Chris Glassey, Robyn Dynes

**Affiliations:** 1 DairyNZ Ltd., Private Bag 3221, Hamilton 3240, New Zealand; alvaro.romera@dairynz.co.nz (A.R.); chris.glassey@dairynz.co.nz (C.G.); 2 AgResearch, Grasslands Research Centre, Palmerston North 4410, New Zealand; kathryn.hutchinson@agresearch.co.nz; 3 AgResearch, Invermay Agricultural Centre, Mosgiel 9053, New Zealand; tony.vanderweerden@agresearch.co.nz (T.v.d.W.); cecile.deklein@agresearch.co.nz (C.d.K.); 4 DairyNZ Ltd., Canterbury Agriculture & Science Centre, Lincoln 7608, New Zealand; dawn.dalley@dairynz.co.nz (D.D.); david.chapman@dairynz.co.nz (D.C.); 5 AgResearch, Lincoln Research Centre, Lincoln 7674, New Zealand; robyn.dynes@agresearch.co.nz

**Keywords:** greenhouse gases, operating profit, mitigations, New Zealand, environmental footprint

## Abstract

**Simple Summary:**

Reducing inputs of nitrogen fertiliser and purchased feed, with an associated reduction in stocking rate on pastoral dairy farms resulted in less nitrate leaching. A co-benefit was a reduction in greenhouse gas emissions. The exception was the implementation of a wintering barn where nitrate leaching was reduced, but greenhouse gas emissions remained unchanged due to greater manure storage and handling. Emission reductions in the lower-input systems came at an average loss of profit of approximately NZ$100 per tonne CO_2_-equivalent.

**Abstract:**

Between 2011 and 2016, small-scale farm trials were run across three dairy regions of New Zealand (Waikato, Canterbury, Otago) to compare the performance of typical regional farm systems with farm systems implementing a combination of mitigation options most suitable to the region. The trials ran for at least three consecutive years with detailed recording of milk production and input costs. Nitrate leaching per hectare of the milking platform (where lactating cows are kept) was estimated using either measurements (suction cups), models, or soil mineral nitrogen measurements. Post-trial, detailed farm information was used in the New Zealand greenhouse gas inventory methodology to calculate the emissions from all sources; dairy platform, dairy support land used for wintering non-lactating cows (where applicable) and replacement stock, and imported supplements. Nitrate leaching was also estimated for the support land and growing of supplements imported from off-farm using the same methods as for the platform. Operating profit (NZ$/ha/year), nitrate leaching (kg N/ha/year), and greenhouse gas emissions (t CO_2_-equivalent/ha/year) were all expressed per hectare of milking platform to enable comparisons across regions. Nitrate leaching mitigations adopted in lower-input (less purchased feed and nitrogen fertiliser) farm systems reduced leaching by 22 to 30 per cent, and greenhouse gas emissions by between nine and 24 per cent. The exception was the wintering barn system in Otago, where nitrate leaching was reduced by 45 per cent, but greenhouse gas emissions were unchanged due to greater manure storage and handling. Important drivers of a lower environmental footprint are reducing nitrogen fertiliser and purchased feed. Their effect is to reduce feed flow through the herd and drive down both greenhouse gas emissions and nitrate leaching. Emission reductions in the lower-input systems of Waikato and Canterbury came at an average loss of profit of approximately NZ$100/t CO_2_-equivalent (three to five per cent of industry-average profit per hectare).

## 1. Introduction

An important challenge facing global dairy industries is to develop farm systems that can maintain or increase production and profitability while reducing environmental impacts, including on water and climate [1,2,3]. Water quality issues have been at the forefront of the environmental concerns in New Zealand (NZ) for a number of decades. More recently, the climate impacts from greenhouse gas (GHG) emissions from agriculture have gained increasing attention. Responding to the effects of anthropogenic GHG emissions on climate, NZ aims to transition to a low-emission economy to help meet the Paris Agreement target of limiting temperature increases to well below 2 °C above pre-industrial levels [4]. New Zealand’s commitment under the Paris Agreement is to reduce GHG emissions by 30% below 2005 levels, by 2030 [5]. In 2017, agriculture was the single biggest contributor (48%) to total GHG emissions in New Zealand, with the dairy sector contributing almost half (47%) of these emissions [6]. The largest sources of agricultural emissions are enteric methane (CH_4_) from ruminant animals and nitrous oxide (N_2_O) emissions from soils.

Although water quality was the focus of much of the environmental research in NZ in recent decades, many of the management practices to improve water quality were also expected to result in reductions in GHG emissions [7]. For example, the Pastoral 21 (P21) research programme [8] included farmlet (small farm) studies in three regions throughout NZ (Waikato, Canterbury, and Otago) that compared systems typical of that region (‘Current’) with ‘Improved’ systems in which strategic changes were made to the Current system. The five key changes used to design the P21 Improved farmlets were using lower nitrogen (N) fertiliser inputs; fewer, but higher producing cows; lower herd replacement rate; greater use of high-energy/low-N feeds; and using off-paddock facilities to reduce the time cows spend on pasture (or on forage crops). In all regions, the Improved systems could reduce nutrient losses to water [8,9,10,11] while GHG emissions were estimated to be reduced in most of the Improved systems [11]. The total annual GHG emissions were strongly related to total feed eaten, and the lower feed supplies and associated lower stocking rates of the Improved systems were the key drivers of lower total GHG emissions in all three regions [11]. These findings align with international studies where the general trend was that increased farming intensity within a system (more input and more animals) may decrease the GHG intensity of milk (kg emissions/kg milk), but absolute emissions (kg emissions/ha) will increase [12,13,14]. Few studies have considered the wider issues of emissions to both air and water, impacts of mitigations on farm profitability, and the potential trade-offs from achieving these often-conflicting goals. The P21 farmlet studies utilised realistic grazing systems and determined both nitrate leaching and GHG emissions as well as systems’ profitability. The aim of this study was, therefore, to analyse the results from these farmlet studies to assess the impact on environmental, production, and economic outcomes of strategies applied to reduce nitrate leaching.

## 2. Materials and Methods

### 2.1. Regional Farmlet Trials

The P21 programme ran small-scale farmlet studies (farmlets ranging from 13 to 39 ha) that included ‘Current’ and ‘Improved’ systems in three regions in New Zealand (Waikato, Canterbury, and Otago; [10]). The ‘Current’ farmlets were designed to represent a system typical of the region in which it was located. The ‘Improved’ farmlets were designed by applying a suite of strategic changes to the Current for each region to reduce nitrate leaching (Table 1). Farm, animal, and feed management practices for the farmlets in each region are described by Clark et al. [8] for Waikato, Chapman et al. [9] for Canterbury, and Van der Weerden et al. [11] for Otago. A summary of the main features is given in Table 2. These farmlets were monitored for production, profitability and nitrate leaching over the following years: 2011 to 2016—Waikato, 2011 to 2014—Canterbury, 2012 to 2015—Otago.

### 2.2. Measuring Production, Nitrate Leaching, and Greenhouse Gases

Individual milk yields (kg milk/cow) were measured for all cows at each milking. Milk component concentrations (MS − milksolids = fat + protein) of both morning and afternoon composite milk samples for each cow were determined weekly for the Waikato farmlets [8] and fortnightly for Canterbury and Otago farmlets [9]. Nitrate leaching from the Waikato farmlets was determined from measurements of nitrate-N concentration in the soil solution below plant rooting depth (collected in porous ceramic cup samplers at a vertical depth of 60 cm). These measurements were used in conjunction with drainage volume (from on-site lysimeters) to estimate leaching losses from the soil [1]. Off-farm sources of nitrate leaching were estimated for fertiliser use for producing pasture for replacement stock, N-excreta deposited by replacement stock, and N fertiliser used for growing imported supplements using the New Zealand Agricultural Inventory methodology (NZAI; [15]). For the Canterbury farmlets nitrate leaching for the milking platform plus winter crop areas was estimated using the Overseer^®^ nutrient budgeting tool (Overseer Limited, Wellington, New Zealand) [9]; Overseer version 6.2.2 was operated using the standard industry operating protocol [16]). Nitrogen loss risk for the Otago farmlets was derived as average values weighted for the respective areas (“blocks”) required for the milking platform, winter and summer forage crops (if needed), young stock rearing, and supplement provision. Estimates of nitrate loss risk were assigned to each of the relevant blocks that made up an individual farmlet. This type of whole-system assessment was based on a combination of directly measured values, proxy values, and literature values [10].

Annual average GHG emissions for each system were estimated for all the monitored years using calculations based on the NZAI methodology (http://www.mfe.govt.nz/node/23304/) [15], but included key farmlet-specific activity data from the P21 farmlet systems as well as farmlet-specific emission factor values determined from targeted regional experiments (see [11] for more detail).

### 2.3. Measuring System Profitability

Operating profit (OP) was determined using a calculator developed specifically for research farmlet trials [17]. This involved scaling the farmlets up to more representative farm sizes for each region (Waikato: 100 ha; Canterbury: 160 ha; Otago: 220 ha), as many farm costs are related to farm and herd size (e.g., labour). Where physical outputs and inputs were known, these were used in the calculation. Where inputs could not be determined separately for each farmlet, average values were used based on regional information from DairyBase (DairyNZ Limited, Hamilton, New Zealand) ([18], a DairyNZ database of farm financial and physical parameters used for benchmarking) and Glassey et al. [19]. A simplified economics model was applied to the biophysical data, using mean values for economically important variables, including supplementary feed and fertiliser prices, and cost data from DairyBase [20] to estimate the profitability of the farmlets. For all profitability calculations, actual milk prices for the monitored years were used. Average milk prices for the monitored years in NZ$/kg MS were Waikato 6.59, Canterbury 7.40, and Otago 7.16. The economic calculations included the cost of rearing replacement stock off-farm [8]. For the Waikato Improved farmlet, the base depreciation rate for capital invested in the off-paddock infrastructure was $350/ha, with an additional $61/ha for maintenance of the infrastructure [8].

## 3. Results and Discussion

### 3.1. Waikato

System changes in the Waikato Improved farmlet resulted in a reduction in nitrate leaching on the milking platform of 23 kg N/ha (equivalent to a 43% reduction) [1]. However, when leaching losses accrued by grazing replacement stock, growing imported supplements, and spreading loafing pad solids off-platform were accounted to the milking platform, the reduction in nitrate leaching was 16 kg N/ha (26% reduction) (Table 3). The collateral benefit of the leaching reduction was a reduction in GHG emissions of 2.2 t carbon dioxide equivalents per hectare (CO_2_-e/ha; 16% reduction). However, the trade-off for the reduced environmental footprint of the Waikato Improved farm had lower production (47 kg MS/ha; 4%) and lower profitability of $280/ha (13%) averaged over five farming seasons (Table 3).

The substantial reduction in profit compared to the relatively small reduction in production can be explained by standing cows off pasture in the Waikato Improved system. Although this mitigation has been confirmed as highly effective for nitrate leaching [1,21,22], the trade-offs are the increase in methane emissions from manure collected in effluent ponds [11,23], and the large costs of the capital investment, depreciation, and maintenance of these facilities [24,25]. The cost of the standing cows off pasture is reflected in other working expenses and overheads and resulted in a 10 cents/kg MS higher cost of milk production (Table 4). Production losses in the Improved system were minimised by using high genetic merit cows achieving high per-cow production, another important driver of efficiency and, therefore, footprint mitigation [26,27], although this target was negated to some extent by an exceptional run of dry years when the desired days in milk for the Improved system could not be achieved [8].

Given the cost of installing and maintaining a stand-off pad in the Waikato, it is worthwhile exploring the potential impact of the multiple system changes where the stand-off approach is excluded. On average, using a stand-off pad would contribute to c. 60% of the nitrate leaching reduction while the ‘low input’ strategies, including higher-producing cows, would contribute c. 40% [1]. The average reduction in nitrate leaching in the Improved system excluding a stand-off pad can, therefore, be estimated as 6 kg N/ha (40% of 16 kg N/ha reduction). By excluding the c. $400/ha cost associated with standing-off, farm profitability in the Improved would be greater than for the Current system. Similarly, by avoiding the increase in net GHG emissions due to the stand-off approach [11], total GHG emissions will be further reduced. This suggests farmers in the Waikato could increase profitability while reducing losses to air and water by implementing a subset of the ‘stacked’ mitigation strategies outlined in Table 1.

The cost of GHG mitigation in the Waikato trial amounted to c. $127/t CO_2_-e at an average milk price of $6.59/kg MS, which can be compared with the cost of $103 and $114/t estimated by Adler et al. [25] for medium input (10%–20% purchased feed) and high input (20%–40% purchased feed) Waikato systems, respectively, using a milk price of $5.50/kg MS. In another study focusing on three Waikato dairy systems (low, medium, high input), Adler et al. [28] estimated the marginal abatement cost for GHG of $96/t CO_2_-e with a $5.50 milk price. In a modelling study of a Waikato dairy system, Smeaton et al. [27] found a weak correlation (R^2^ = 0.43) between GHG emissions and profitability with an average abatement cost of c. $250/t CO_2_-e. Carbon prices are rising [29], and about half of the global GHG emissions are now covered by carbon pricing initiatives priced at over US$10/t CO_2_-e (~NZ$15) compared with one-quarter of emissions covered in 2017. It is clear that carbon prices will have to increase substantially before it is economically worthwhile for dairy farmers to adjust their system instead of offsetting emissions by buying carbon credits (note: agriculture is currently not included in New Zealand Emissions Trading Scheme). However, the situation may change in the not too distant future if we consider that the High-Level Commission on Carbon Prices identified the carbon price to be in the range of US$40–80/t CO_2_-e in 2020 and US$50–100/t CO_2_-e by 2030, which will make it consistent with achieving the temperature goal of the Paris Agreement [29]. To shift investment at scale, carbon pricing coverage must expand, and prices must be stronger. Most initiatives saw increases in carbon prices in 2018 compared to price levels in 2017. However, despite these, most initiatives in 2019 are still below the US$40–$80/t CO_2_-e needed in 2020 [29].

Compared with commercial farms in the Waikato region, the Current farm performed well above average in terms of production and profit (Table 3 and Table 5), and it was clearly not an average or typical farm. The reasons could be the environmental conditions and/or measurement and managerial intensity applied at the research site. In the context of “average” commercial farms, the Waikato Improved system shows a lot of potential by maintaining production, trading a relatively small amount of profit, and leaving a modest environmental footprint. However, it should be considered that the gains made on the trial farms were made by running the farms with best-management practices, smaller reductions at higher profit trade-offs may be expected from most commercial farms.

### 3.2. Canterbury

In the Canterbury region, the Improved system reduced nitrate leaching from the milking platform by 14 kg N/ha (30% reduction) compared with the Current system [9]. When including nitrate leaching losses from the winter crop, the reductions in the Improved system were 22 kg N/ha (29%) with fodder beet and 34 kg N/ha (30%) with kale (Table 3). Leaching from both these winter crops was generally high (150–200 kg N/ha crop), but the larger area required for the lower-yielding kale crop resulted in higher nitrate leaching losses per hectare of platform area, compared with fodder beet. The co-benefit of the lower leaching in the Improved system was a reduction in GHG emissions of 5 t CO_2_-e/ha (24%) compared with the Current. However, trade-offs of the Improved system were reductions in production (minus 542 kg MS/ha, 24%) and profit (minus $358/ha, 9%). The cost of GHG abatement was $71/t CO_2_-e, which is much lower than for the Waikato but still substantially higher than the current carbon price.

The operating profit for both Canterbury systems were higher than the average of surrounding commercial farms, mainly driven by higher production (Table 3 and Table 5). Operating expenses for the trial farms ($4.7 and $4.4/kg MS for Current and Improved, respectively) were also lower than for the commercial farms (Table 4). The evidence from the Improved farmlet demonstrates that there are system options that Canterbury farmers could adopt to reduce their environmental footprint. Already, the Lincoln University Dairy Farm has successfully adopted the P21 Improved system at a whole farm scale [30]. There will be trade-offs compared to best-practice current systems, but with efficiency gains, both production and profit can be above the current averages for the region. Such efficiency gains will require improved management ability and processes on farms. This is important information for building farmer confidence in the face of regulatory change [9].

### 3.3. Otago

Two Improved systems were tested in Otago, one with duration-controlled grazing, where cows were housed in a barn for 12 h/day on wet days in spring and autumn and 24 h/day in winter from June to mid-August to reduce urinary N deposition onto wet soils (Improved-barn), and one attempting to optimise feed intake by changing calving date and type of home-grown feed (Improved-opt). Both Improved systems used less N fertiliser (Table 2). Leaching was reduced by 13 kg N/ha (45%) and 7 kg N/ha (24%) in the Improved-barn and Improved-opt systems, respectively, compared with the Current system (Table 3). A collateral benefit was GHG reductions of 0.3 (3%) and 1.1 (9%) t CO_2_-e/ha from the barn and optimal-feeding systems, respectively. The small reduction in GHG emissions from the barn system was the result of an increase in the amount of manure that required active management with associated GHG emissions, which largely negated the gains made by reducing urinary N onto wet soils. Van der Weerden et al. [11] showed that off-paddock facilities can increase emissions per cow from manure management, with the magnitude of the increase depending on the extent of the facility’s use. For the Otago situation, the use of the barn for 24 h/day in winter and 12 h/day on wet days in autumn and spring corresponded to a 35% increase in GHG emissions per cow. For both Otago Improved systems trade-offs in production were small at −15 (2%) and −33 (3%) kg MS/ha for the barn and optimal-feeding systems. However, the profitability of the barn system was significantly lower (−NZ$700/ha). This was mainly due to extra depreciation on the capital required for the barn itself, the effluent spreader, and extra silage bunker space (Overheads, Table 4). Maintenance costs were also higher because of the need to deal with more captured effluent and the cost of replacing the woodchip bedding for the barn (other working expenses, Table 4). The average profit in the optimal-feeding system was moderately higher (NZ$62/ha) compared with the Current system, mainly because of lower feed and fertiliser expenses (Table 4).

Compared with commercial farms in the Otago region (Table 5), the profitability of all systems was considerably lower (Table 3). The main contributor to the higher operating expenses/kg MS was the poor MS production/ha across all systems. Factors that contributed to low MS production/ha included the below-average genetic merit of the herd, a third of the farm being a recent conversion from sheep farming without renovating the poor-quality sheep pastures and upgrading the water supply system, drainage issues on the low lying heavier soils and the geographical spread of the farm resulting in increased energy expenditure and lameness from long walks on undulating terrain. The complex management structure of the property meant the business was not as agile at responding to climatic challenges and market signals as commercial businesses in the region, which impacted on the physical and financial performance of the farm.

### 3.4. Insights across Regions

Greenhouse gas reductions from lower-input, lower-stocked systems in the Waikato and Canterbury regions came at an average loss of profit of approximately NZ$100/t CO_2_-e. This mitigation cost needs to be viewed in the context of on-farm forestry that can achieve the largest emission reductions (3–96%), depending on the percentage of the land planted. However, this is an expensive option for dairy farms with an implied C cost in excess of NZ$100–600/t CO_2_-e, mainly because of the large opportunity cost incurred when taking land out of dairy grazing. The most viable option for dairy farms would be forests planted only on marginal land and not for harvest, which depend heavily on individual farm configurations and has a more limited mitigation potential of up to 10% of emissions [5].

Analysis of the Waikato Improved system without the loafing pad pointed to a profitable system that can achieve nitrate leaching and GHG mitigations without requiring extra investment in infrastructure. The potential for environmental mitigation without infrastructure and without sacrificing profitability was further supported by the results from the Otago Improved-opt system. This is relevant to many farm systems that are starting from a low baseline where extra investment and/or lower profitability is simply out of the question. These systems can benefit from gradually improving the genetic merit of their herds over time.

The positive relationship between nitrate leaching and GHG emissions observed in Waikato and Canterbury agrees with previous works [22,27,31] and confirms the potential positive by-product of nitrate leaching regulation on GHG emissions. Two drivers of the lower environmental footprint of the Waikato and Canterbury Improved systems were lower N fertiliser use and lower stocking rate, which agree with the findings of several studies that these are key factors in pasture-based dairy systems determining the balance between production, profit, and environmental footprint [25,26,27,28,32,33,34].

## 4. Conclusions

Important drivers of a lower environmental footprint (GHG emissions and nitrate leaching) are reducing nitrogen fertiliser and purchased feed. This reduces nitrogen surplus and feed flow through the herd and drives down both GHG emissions and nitrate leaching. Nitrate leaching mitigations in the P21 farmlet systems achieved leaching reductions of 24 to 30 per cent. In addition, these lower-input (less purchased feed and N fertiliser) systems also reduced GHG emissions by between 9% and 24%. The exception was the Improved-barn system in Otago, where nitrate leaching was reduced by 45 per cent but GHG emissions were not reduced due to greater manure storage and handling. Greenhouse gas reductions in the lower input systems of Waikato and Canterbury came at an average loss of profit of approximately NZ$100/t CO_2_-e (three to five per cent of industry-average profit per hectare). Economic impacts of Improved systems were highly regional specific and highlighted the need for future systems to perform better than current local systems, requiring strong management expertise, with consideration for investment in infrastructure. However, for system changes that do not include infrastructure investment, profitability can increase while associated losses to air and water decrease.

## Figures and Tables

**Table 1:** System changes applied to typical regional dairy farms in developing nitrate leaching mitigated farms as part of the Pastoral 21 farmlet trials in Waikato, Canterbury, and Otago, New Zealand [11].

Region	Fewer, Higher Producing Cows	Reduced N Fertiliser Inputs	Reduced Herd Replacement Rate	Greater Use of High Energy/Low N Feeds	Off-Paddock Facilities
Waikato	√	√	√	√	√
Canterbury	√	√		√	
Otago		√	√		√

**Table 2:** Key management features of control (Current) and improved systems (Improved) in Waikato, Canterbury, and Otago; opt = optimised feeding; barn = cows housed during winter and some wet days in autumn and spring. Adapted from Van der Weerden et al. [11].

System Feature	Waikato		Canterbury		Otago		
	Current	Improved	Current	Improved	Current	Improved-Opt	Improved-Barn
Stocking rate (cows/ha)	3.2	2.6	5.0	3.5	3.0	2.8	2.8
Cow genetic merit ($BW^#^)	90	170	133	140	109	105	104
N fertiliser (kg N/ha/year)	137	52	311	158	109	42	73
Replacement rate (%)	22	18	23	23	23	18	18
High energy/low N feed	N/A	0.24 (Grain t DM/cow/year)	N/A	40% diverse pasture	N/A	N/A	N/A
Stand-off/housing	No	Yes	No	No	No	No	Yes
Winter feed	On platform	On platform	Fodder beet + Pasture silage	Kale + Oat silage	Kale	Kale	N/A
N fertiliser for winter forage (kg N/ha/year)	N/A	N/A	200	307	200	200	N/A

N/A: not applicable; ^#^ Breeding worth, $ (May 2011).

**Table 3:** Average performance (production, profit, and environmental losses) of three regional farm system trials. All metrics are presented as per hectare of the milking platform. Numbers in brackets indicate the range for all farming seasons available. In the Canterbury region, wintering of non-lactating cows can be either on kale followed by an oats catch crop (Kale), or fodder beet (FB). Greenhouse gas data from [11].

Region	Farm System	Milk Production (kg MS/ha)	Operating Profit ($/ha)	Nitrate Leaching (kg N/ha)	Greenhouse Gas (t CO_2_-e/ha)
Waikato	Current	1200 (1151 to 1232)	2086 (−244 to 3873)	62 (43 to 75)	13.61
Waikato	Improved	1153 (1093 to 1207)	1807 (−834 to 3652)	46 (37 to 57)	11.405
Canterbury	Current	2242 (1834 to 2428)	3893 (3596 to 4440)	Kale: 114 FB: 75	20.615
Canterbury	Improved	1700 (1452 to 1808)	3535 (3283 to 3885)	Kale: 80 FB: 53	15.582
Otago	Current	964 (915 to 1040)	715 (−1428 to 3226)	29 (24 to 38)	11.827
Otago	Improved-barn	949 (913 to 983)	20 (−1980 to 2473)	16 (10 to 22)	11.461
Otago	Improved-opt	931 (899 to 969)	777 (−1192 to 3040)	22 (15 to 31)	10.792

**Table 4:** Average financial results of the Pastoral 21 regional farm trials. All results are expressed per hectare of the milking platform. Numbers in brackets indicate the range for all farming seasons available.

Region	Waikato	Waikato	Canterbury	Canterbury	Otago	Otago	Otago
Farm System	Current	Improved	Current	Improved	Current	Improved-Barn	Improved-Opt
Dairy gross farm revenue ($/ha)	7713 (5260 to 9702)	7363 (4670 to 9352)	15,305 (15,081 to 15,510)	11,445 (11,357 to 11,656)	6671 (4748 to 9216)	6430 (4710 to 8652)	6349 (4463 to 8565)
Total feed expenses ($/ha)	965 (804 to 1179)	923 (719 to 1163)	4324 (3831 to 4657)	2208 (1995 to 2422)	1729 (1572 to 1950)	1458 (1269 to 1618)	1539 (1480 to 1629)
Total stock expenses ($/ha)	745 (720 to 771)	632 (614 to 644)	1379 (1,369 to 1,387)	972 (970 to 978)	645 (606 to 666)	624 (617 to 638)	609 (600 to 617)
Total labour expenses ($/ha)	1079 (1079 to 1079)	1026 (1026 to 1026)	1554 (1554 to 1554)	1554 (1554 to 1554)	1043 (1030 to 1052)	1036 (1034 to 1041)	1034 (1034 to 1034)
Total other working expenses ($/ha)	1858 (1798 to 1884)	1924 (1803 to 2140)	3409 (3,353 to 3446)	2479 (2468 to 2502)	1523 (1478 to 1602)	1901 (1852 to 1961)	1381 (1361 to 1412)
Total overheads ($/ha)	980 (979 to 981)	1051 (1050 to 1052)	746 (742 to 750)	697 (695 to 699)	1014 (1002 to 1035)	1391 (1369 to 1432)	1010 (982 to 1024)
Dairy operating expenses ($/ha)	5628 (5495 to 5829)	5556 (5457 to 5700)	11,412 (10,926 to 11,775)	7910 (7682 to 8113)	5955 (5701 to 6175)	6411 (6179 to 6690)	5572 (5525 to 5655)
Operating expenses ($/kg MS)	4.7 (4.5 to 4.8)	4.8 (4.6 to 5)	4.7 (4.5 to 4.9)	4.4 (4.3 to 4.6)	6.2 (5.8 to 6.6)	6.8 (6.3 to 7)	6 (5.7 to 6.3)
Dairy operating profit ($/ha)	2086 (−244 to 3873)	1807 (−834 to 3652)	3893 (3596 to 4440)	3535 (3283 to 3885)	715 (−1428 to 3226)	20 (−1980 to 2473)	777 (−1192 to 3040)

**Table 5:** Average performances of typical commercial dairy farms in the same regions as the P21 farmlet trials. Extracted for the relevant years from DairyNZ Economic Survey data (https://www.dairynz.co.nz/publications/dairy-industry/).

Performance Indicators	Waikato 2011–2016	Canterbury 2011–2014	Otago 2012–2015
Number of herds	56	23	28
Peak cows	343	751	587
Effective hectares	120	222	209
Stocking rate (cows/ha)	2.8	3.4	2.8
Milk production (kg MS/ha)	1025	1413	1120
Milk price ($/kg MS)	6.59	7.40	7.16
Operating expenses ($/kg MS)	4.80	4.96	4.95
Operating profit ($/ha)	1949	3438	2505
